# Differential Anti-Glycan Antibody Responses in *Schistosoma mansoni*-Infected Children and Adults Studied by Shotgun Glycan Microarray

**DOI:** 10.1371/journal.pntd.0001922

**Published:** 2012-11-29

**Authors:** Angela van Diepen, Cornelis H. Smit, Loes van Egmond, Narcis B. Kabatereine, Angela Pinot de Moira, David W. Dunne, Cornelis H. Hokke

**Affiliations:** 1 Department of Parasitology, Center of Infectious Diseases, Leiden University Medical Center, Leiden, The Netherlands; 2 Vector Control Division, Ministry of Health, Kampala, Uganda; 3 Department of Pathology, University of Cambridge, Cambridge, United Kingdom; Federal University of Minas Gerais, Brazil

## Abstract

**Background:**

Schistosomiasis (bilharzia) is a chronic and potentially deadly parasitic disease that affects millions of people in (sub)tropical areas. An important partial immunity to *Schistosoma* infections does develop in disease endemic areas, but this takes many years of exposure and maturation of the immune system. Therefore, children are far more susceptible to re-infection after treatment than older children and adults. This age-dependent immunity or susceptibility to re-infection has been shown to be associated with specific antibody and T cell responses. Many antibodies generated during *Schistosoma* infection are directed against the numerous glycans expressed by *Schistosoma*. The nature of glycan epitopes recognized by antibodies in natural schistosomiasis infection serum is largely unknown.

**Methodology/Principal Findings:**

The binding of serum antibodies to glycans can be analyzed efficiently and quantitatively using glycan microarray approaches. Very small amounts of a large number of glycans are presented on a solid surface allowing binding properties of various glycan binding proteins to be tested. We have generated a so-called shotgun glycan microarray containing natural N-glycan and lipid-glycan fractions derived from 4 different life stages of *S. mansoni* and applied this array to the analysis of IgG and IgM antibodies in sera from children and adults living in an endemic area. This resulted in the identification of differential glycan recognition profiles characteristic for the two different age groups, possibly reflecting differences in age or differences in length of exposure or infection.

**Conclusions/Significance:**

Using the shotgun glycan microarray approach to study antibody response profiles against schistosome-derived glycan elements, we have defined groups of infected individuals as well as glycan element clusters to which antibody responses are directed in *S. mansoni* infections. These findings are significant for further exploration of *Schistosoma* glycan antigens in relation to immunity.

## Author Summary

Schistosomes are parasitic worms that cause chronic and potentially deadly disease in millions of people in (sub)tropical areas. An important partial immunity to infection does develop but this takes many years of exposure and multiple infections. Therefore, children are far more susceptible to re-infection after treatment than adults. This immunological protection is associated with specific antibody and T cell responses. Many antibodies generated during *Schistosoma* infection are directed against carbohydrate chains (glycans) expressed by the parasite. The nature of the glycan epitopes recognized by antibodies in natural schistosomiasis infection serum is largely unknown. We have used a so-called shotgun glycan microarray approach to study differences in anti-glycan antibody responses between *S. mansoni*-infected children and adults. This resulted in the identification of differential glycan recognition profiles characteristic for the two different age groups that may reflect differences in age or differences in length of exposure or infection in people living in an endemic area.

## Introduction

Schistosomiasis (bilharzia) is a chronic and potentially deadly parasitic disease, and a major public health burden in (sub)tropical areas. An estimated 207 million people are affected and 779 million people are at risk of being infected with schistosomes [1], [2]. Schistosomiasis is caused by members of the helminth genus *Schistosoma* (*S.*) with *S. haematobium, S. mansoni, and S. japonicum* being the most widespread. Schistosomes have a complex life-cycle with larval, adult worm, and egg stages interacting with the human host, each playing a role in immunology, immunopathology and maintenance of infection. *Schistosoma* infection is commonly treated with Praziquantel (PZQ) [3], [4]. Although PZQ has proven to be very effective, concern has been raised about development of drug resistance upon the currently ongoing mass treatments in endemic areas [5], [6] and the need for an alternative anti-schistosomal drug is regularly emphasized [7]. Furthermore, drug treatment does not prevent reinfection and repeated treatments are essential for people living in endemic areas, resulting in high costs and requirements to infrastructure. Therefore it is of great importance that a vaccine inducing protection against schistosomiasis is developed.

Multiple longitudinal studies have shown that infected individuals do acquire significant levels of immunity after prolonged exposure to *Schistosoma*. The acquisition of immunity is age-dependent in human populations living in schistosomiasis endemic areas with children being far more susceptible to re-infection than older children and adults [8]–[12] indicating that it takes many years of exposure, multiple infections and treatments, and maturation of the immune system to acquire this type of immunity. Several immunological parameters, including specific antibody and T cell responses, are predictive of the age-dependent immunity or susceptibility to re-infection after treatment [8], [13], [14]. Especially high levels of IgE against adult worm antigens [15]–[20], but also IgG1, IgG3 and IgA [8], [14] levels have been associated with increased resistance to infection after treatment. IgM, IgG2 and IgG4, on the other hand, are blocking antibodies with possible detrimental consequences for the expression of protective immunity [14], [21]. IgM can block eosinophil-dependent killing mediated by IgG antibodies from the same or other sera [22], [23]. IgM was found to be more highly expressed in children than in adults and is therefore higher in the non-immune group compared to the more resistant people [24], [25].

Antibody responses in schistosomiasis have been mainly studied using soluble worm antigen (SWA) and soluble egg antigen (SEA), each consisting of complex mixtures of antigenic (glyco-)proteins, or using specific recombinant protein antigens. Most antibodies generated during *Schistosoma* infection are however directed against parasite glycans [26]–[30]. This is not surprising considering the fact that glycans are abundant in schistosomal secretions, decorate the outer surface of all *Schistosoma* stages, and are highly immunogenic [31], [32]. *Schistosoma* life stages each express a different glycan repertoire [31], [33], [34]. Elaborate studies on the glycome of the different *Schistosoma* life stages have indicated that hundreds of different glycan structures are present within the N- and O-linked glycans and the glycolipids [31]. So far, serum antibodies to only a small set of schistosome-related glycans have been determined in a limited number of studies [25], [29], [30]. The large gap in our knowledge about the contribution of anti-glycan antibodies to immunity to schistosomes may be overcome using a shotgun glycan array approach which allows the detection of serum antibodies to a large number of parasite-derived glycans simultaneously. In this glycan array technology, natural glycans isolated directly from relevant cells or organisms are presented on a surface to quantitatively measure the binding to complementary molecules at the whole natural glycome level thus including unique and unusual (e.g. pathogen-specific) glycans [1], [35]–[40]. We have generated such a shotgun glycan microarray containing natural N-glycan and lipid-glycan fractions derived from 4 different life stages of *S. mansoni* (male adult worm, female adult worm, cercariae, and eggs), and applied this array to the analysis of IgG and IgM serum antibodies in a selection of sera from an *S. mansoni* natural infection cohort. This resulted in the identification of antigenic glycans as well as differential glycan recognition profiles characteristic for different age groups and shows that the shotgun schistosome glycan microarray approach has discriminative properties to define groups of infected individuals.

## Methods

### Ethics statement

Ethical approval for the Piida study was obtained from the Uganda National Council for Science and Technology (UNCST) and cleared by the Office of the President. The study was also supported by the Cambridge Local Research Ethics Committee. Prior to enrolment, the study was explained to each selected adult or parent/guardian of each selected child for the study and verbal consent obtained. Verbal informed consent was sought because of the high level of illiteracy in Piida and because Lougungu, the predominant language, is not a written language. This method was approved by the ethical review committee of the UNCST. Verbal consent was documented by recording the name of each individual providing consent.

### Materials

*S. mansoni* adult worms, cercariae and eggs were obtained as reported previously (Robijn et al, 2005). BSA- and NH_2_-linked synthetic oligosaccharide conjugates were synthesized as described [35], [41]–[44]. Cy3 conjugated goat anti-human IgG (Fc-specific), BSA and ethanolamine were from Sigma (Zwijndrecht, the Netherlands). Alexa fluor 647 conjugated goat anti-human IgM (μ chain specific) was from Invitrogen (Breda, The Netherlands).

### Sera

Human sera were obtained from *S. mansoni* infected individuals living in the Piida community, Butiaba, which is situated on the shore of Lake Albert in Uganda where *S. mansoni* is endemic with 72% prevalence [24], [45], [46]. The detection of *S. mansoni* eggs in the feces was used as an indicator of infection with *S. mansoni*. The study design, epidemiology, and sample collection have been described in detail previously [24]. In the current study, anti-glycan antibody responses were determined among two separate age-groups, 21 children aged 5–11 years (mean age: 9) and 20 adults aged 20–46 years (mean age: 29), non-randomly selected from the original Piida study cohort based on intensity of infection and sex. All subjects had patent *S. mansoni* infection and intensity of infection did not differ significantly between the two groups [*P* = 0.51, geometric mean (GM) infection intensity (epg) was 478.33 (CI_95%_: 260.90, 868.37) among children and 665.80 (CI_95%_: 278.39, 1592.36) among adults]. The two groups were comparable with respect to sex, with roughly 3 females: 2 males in both age-groups. Anti-SEA-IgG_4_ and -IgE and anti-SWA-IgG_4_ responses were comparable in the two age groups (*P*>0.20); anti-SEA-IgG_1_ responses were significantly greater among the children (*P*<0.001), whilst anti-SWA-IgG_1_ and -IgE were significantly greater among the adults (*P*≤0.01).

### Glycan release

*S. mansoni* male and female worms, cercariae and eggs were homogenized in water (4 ml per g wet weight) and sequentially methanol and chloroform were added (7 and 13 volumes, respectively). The upper phase contains the glycolipids and the pellet the (glyco)proteins. Glycans were released from the different preparations of *S. mansoni* glycolipids and glycoproteins by ceramidase and PNGase F treatment, respectively. Released glycans were subsequently purified, labeled with 2-aminobenzoic acid (2-AA), and fractionated by hydrophobic interaction liquid chromatography with fluorescence detection, as described previously [35], [47].

### Glycan microarray construction

Glycan fractions, (synthetic) glycoconjugates, and proteins were dissolved in 20 µl of 1× spotting buffer (Nexterion Spot, Schott Nexterion) with 10% DMSO in 384-wells V-bottom plates (Genetix, New Milton, UK). A total number of 1143 samples (192 from male worms, 192 from female worms, 384 from cercarial lipid glycans, 192 from cercarial N-glycans, 102 from egg N-glycans, and 81 (synthetic) glycoconjugates) were printed in triplicate on epoxysilane-coated glass slides (Slide E, Schott, Nexterion) by contact printing using the Omnigrid 100 microarrayer (Genomic Solutions, Ann Arbor, MI) equipped with SMP3 pins with uptake channels that deposit 0.7 nl at each contact. Each array was printed three times on each glass slide. Dot spacing was 290 µm (X) and 245 µm (Y), and array spacing was 6000 µm. Printed slides were incubated overnight at room temperature at sufficient humidity to prevent drying of the spots and to allow covalent binding of printed 2-AA-labeled glycans and glycoconjugates to the epoxysilane via reaction with primary or secondary amines [35].

### Binding assay

Microarray slides were covered with a hand-cut silicone gasket creating barriers to separate the three printed arrays and to hold wash and incubation solutions within the individual array areas. To remove unbound compounds, the arrays were rinsed with 1 ml PBS. Remaining active epoxysilane groups were blocked with 2% BSA, 50 mM ethanolamine in PBS for 60 minutes at room temperature while shaking. Subsequently, the slides were rinsed with PBS. Each microarray was incubated with serum (diluted 1∶100 in PBS-0.01% Tween20 with 1% BSA) for 60 min at room temperature while shaking. After washing the slides with successive rinses of PBS-0.05% Tween20 and PBS, the slides were incubated with Cy3-labeled anti-human IgG and Alexa Fluor 647-labeled anti-human IgM (diluted 1∶1,000 in PBS-0.01 Tween20) for 30 minutes at room temperature while shaking and protected from exposure to light. After a final rinse with PBS-0.05% Tween20, PBS and water the slides were dried and kept in the dark until scanning.

### Scanning and data analysis

A G2565BA scanner (Agilent Technologies, Santa Clara, CA) was used to scan the slides for fluorescence at 10 µm resolution using 2 lasers (532 nm and 633 nm). At these wavelengths the 2-AA label does not fluoresce. Data and image analysis was performed with GenePix Pro 6.0 software (Molecular Devices, Sunnyvale, CA). Spots were aligned and re-sized using round features with no CPI threshold. Background-subtracted median intensities were averaged and processed as described by Oyelaran et al. [48] and median values of negative controls included on each array were subtracted. Datasets were log_2_ transformed to remove the basic trends of variance and plotted against the sample numbers. Hierarchical clustering analysis (HCA, complete linkage clustering using Euclidean distance) and Principal component analysis (PCA) were performed to define associated groups of sera and glycan fractions using MultiExperiment Viewer v4.5 and Simca-P+ 12.0 (Umetrics), respectively. For HCA, non-parametric testing was used for comparisons and a p value <0.01 was used to identify glycan fractions that were differentially recognized by serum antibodies [49].

### Mass spectrometry

Glycan samples of interest were analyzed by matrix-assisted laser desorption ionization time of flight mass spectrometry (MALDI-TOF-MS) with an Ultraflex II mass spectrometer (Bruker Daltonics, Bremen, Germany) in the negative ion reflectron mode using 2,5-dihydroxybenzoic acid (DHB, Bruker Daltonics) (20 mg/ml in 30% ACN) as matrix. Glycopeakfinder (http://www.glyco-peakfinder.org) was used to define glycan composition.

## Results

### Age group comparisons

**Figure 1:**
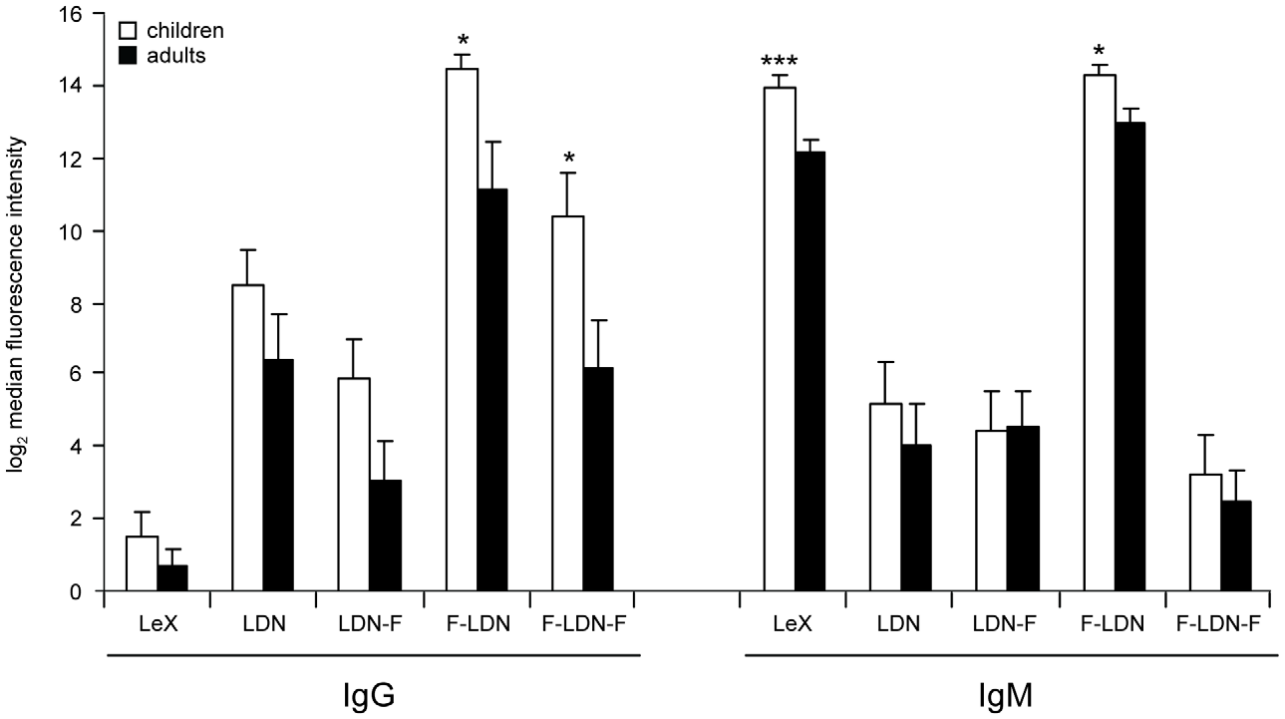
Comparison of antibody responses to glycoconjugates in young and older age groups. Binding of IgG and IgM in sera from *S. mansoni* infected children (white bars) and adults (black bars) to a restricted set of BSA conjugated synthetic glycan structures. Average log_2_ transformed background subtracted median fluorescence intensities are shown.

Using the shotgun glycan microarray, anti-glycan IgG and IgM responses in sera from *S. mansoni* infected individuals were determined. First, the IgG and IgM responses against a set of BSA-conjugated synthetic glycan structures that were included in the glycan microarray were compared between the two age groups (<12 years vs >20 years). Overall, the IgG response was higher in the group of children compared to adults with significant differences between the groups in response to Fuc(α1–3)GalNAc(β1–4)GlcNAc (F-LDN) and Fuc(α1–3)GalNAc(β1–4)[Fuc(α1–3)]GlcNAc (F-LDN-F) (Figure 1). Also the IgM response was higher in children and differed significantly from that in adults for Gal(β1–4)[Fuc(α1–3)]GlcNAc (Lewis X, LeX) and F-LDN (Figure 1). When comparing IgG and IgM responses, IgG responses against F-LDN-F and GalNAc(β1–4)GlcNAc (LDN) were significantly higher than IgM in both age groups, while responses against LeX were dominated by IgM (Figure 1).

**Figure 2:**
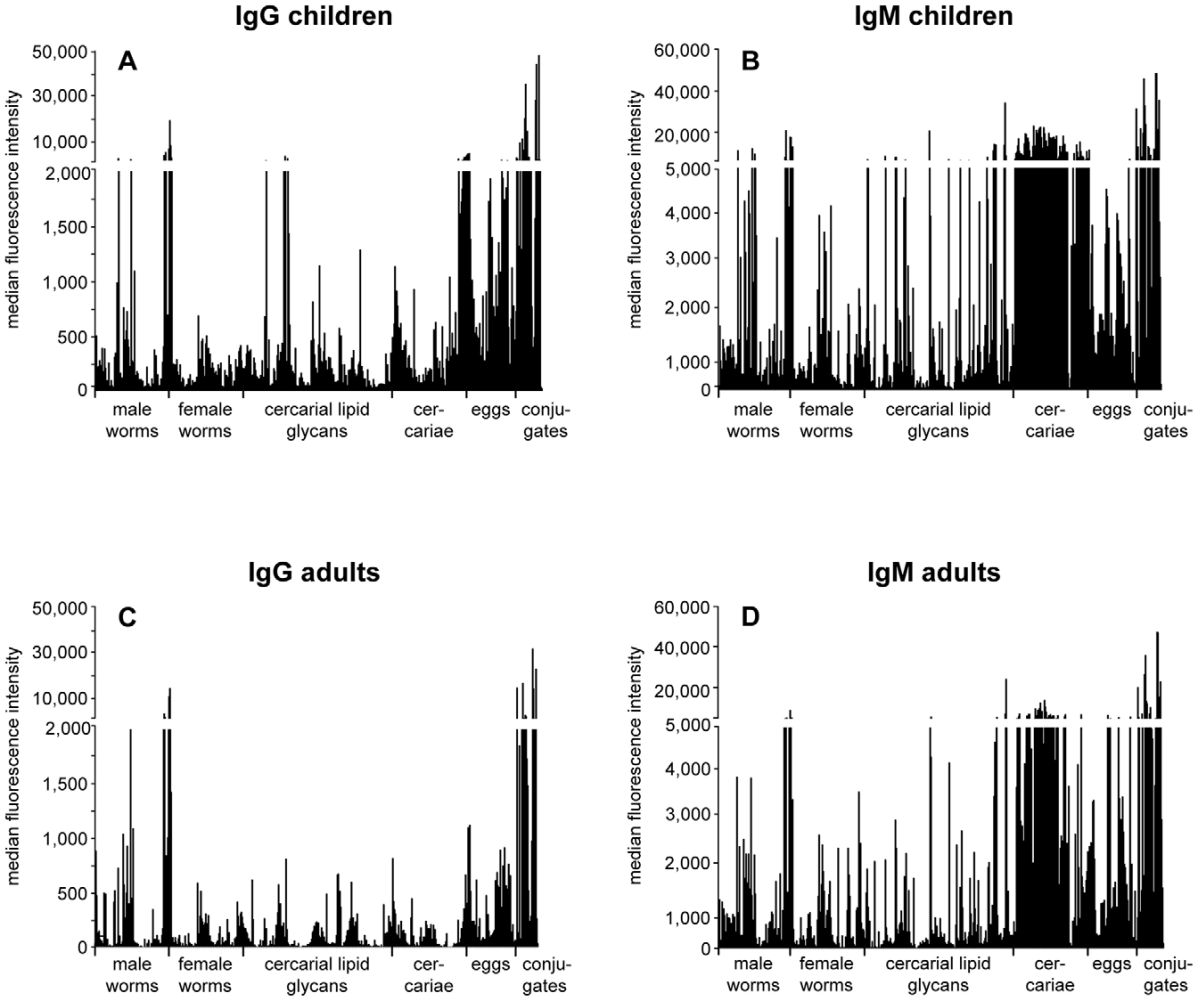
Shotgun glycan microarray. Binding of serum antibodies from *S. mansoni* infected individuals <12 years (A and B) and >20 years (C and D) to glycan fractions from different life stages of *S. mansoni*. Average background subtracted median fluorescence intensities are shown for IgG (A and C) and IgM (B and D).

**Figure 3:**
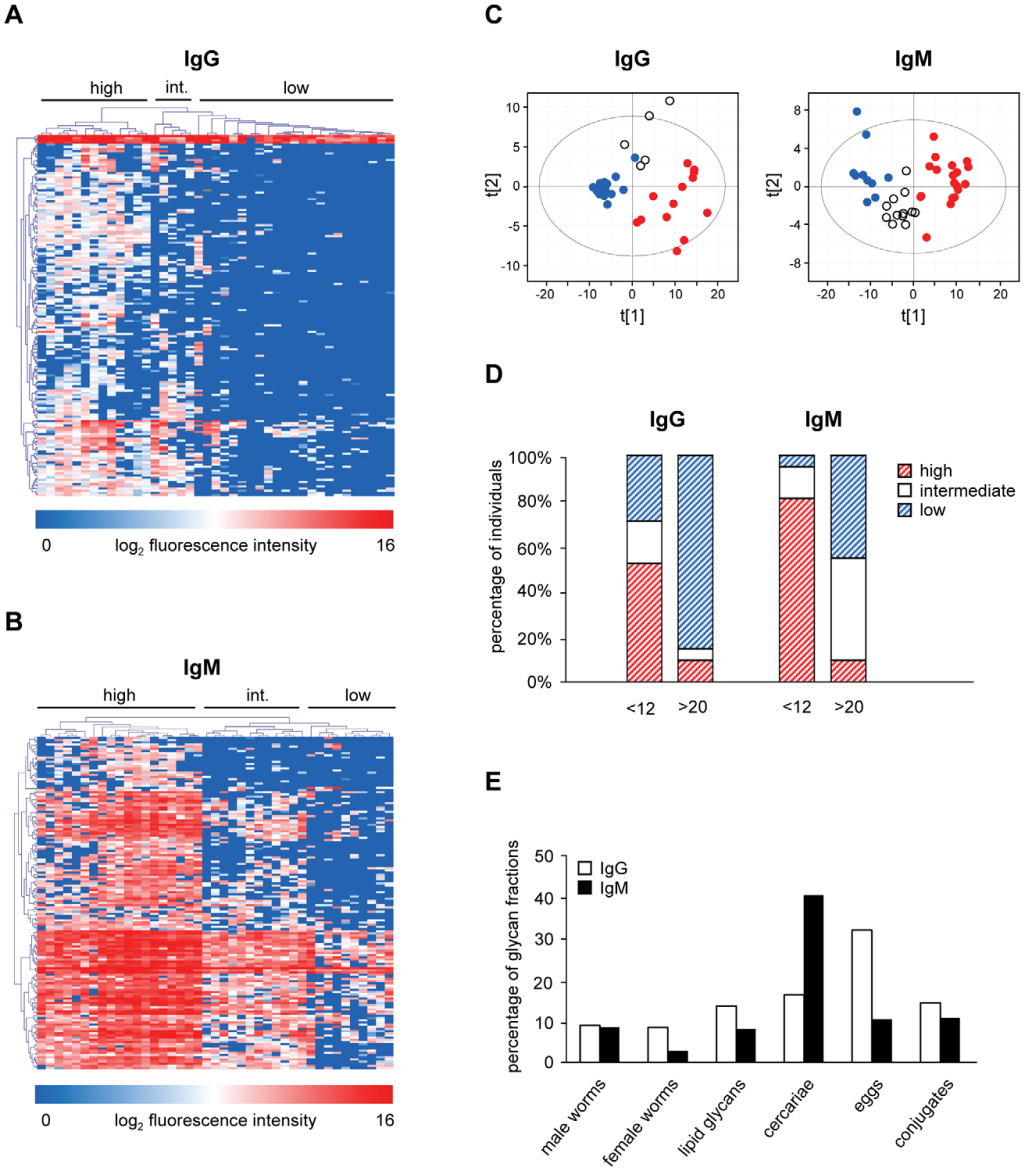
Age groups. Comparisons of anti-glycan responses between age group <12 years and >20 years. Supervised HCA and PCA of differentially expressed glycan fractions are shown for IgG (A+C) and IgM (B+C), percentage of individuals per age group clustering together in the supervised HCA (D) and percentage of differentially recognized glycan fractions (E). For HCA, individuals are reported in horizontal dimension and differentially recognized glycan fractions in the vertical dimension. In (C) and (D), individuals clustering in high, intermediate (int.), and low response clusters are represented by red, white, and blue dots and (dashed) bars, respectively. In (E), the number of differentially recognized glycan fractions is depicted as a percentage of the total number of glycan fractions from each source. White and black bars represent results for IgG and IgM respectively.

With respect to the numerous printed glycans isolated directly from the *Schistosoma* life stages, Figure 2 shows that overall the IgG and IgM response patterns against the different glycan fractions are similar between the two age groups, but with a higher anti-glycan response intensity in the age group <12 years. Examining the responses against individual glycan fractions printed, statistical analysis using a Mann Whitney U rank order test (p<0.01) revealed a significant difference between the two age groups for 14.5% and 13.4% of all glycan fractions present on the array for IgG and IgM respectively with all responses being higher in children than in adults. For IgG, this group of differentially recognized glycans mainly consisted of cercarial glycolipid glycans (n = 54), cercarial (n = 32) and egg N-glycans (n = 33), while for IgM the differentiating fractions contained glycans isolated from cercariae (N-glycans followed by lipid glycans, n = 78 and 32 respectively). Since the number of glycan fractions printed on the array was not equal for all sources, the numbers of differentially recognized glycan fractions were plotted as percentages of the total number of glycan fractions from each source (Figure 3E). This showed that almost one third (32.4%) of the total number of egg-derived N glycans were differentially recognized by IgG when comparing the responses between children and adults, while for IgM this was highest for the cercarial N-glycans (40.6%). To explore which glycan structures were differentially recognized between children and adults, the top 10 of glycan fractions with the biggest difference in response were analyzed by MALDI-ToF-MS (Tables S1 (IgG) and S2 (IgM)). Most of these fractions contained mixtures of glycans, and of potential antigenic glycan elements. The glycan fractions that were differentially recognized by IgM and were higher in children than in adults contained glycans with short fucosylated and/or xylosylated (truncated) core structures and a few more complex structures which contain both core fucose and xylose and LeX elements in the antennae. For IgG, the proposed glycan structures are more complex and may contain other types of glycan elements such as LeX-LeX (di-LeX) and GlcNAc-LeX (extended LeX). HCA and PCA of the subset of differentially recognized glycan fractions between the two age groups showed three clusters for IgG (high (red), intermediate (white), and low response (blue)) (Figures 3A and 3C). For the group <12 years, 11 individuals (52.4%) clustered together in the high response cluster, 4 children clustered in the intermediate (19%) and 6 (28.6%) in the low response group (Figure 3D). In contrast, only 3 adults showed a high or intermediate IgG response while the majority shows a low (85%) IgG mediated response (Figure 3D). Three clusters were observed for IgM (high, intermediate and low response) (Figures 3B and 3C). Most of the children (81%) clustered in the high response cluster while the majority of adults clustered in the low (45%) and intermediate (45%) response clusters (Figure 3D). These data indicate that IgG and IgM responses can be different for a selection of individuals since some of the high IgM responders did not show a high IgG mediated response. All of the children clustering in the high IgG response cluster also showed a high IgM response.

### Non-supervised comparisons–clustering of individuals

The results described above show that, although the responses are significantly different between the two age groups, the individuals do not cluster precisely according to the age groups. Especially the intermediate response clusters contain individuals from both age groups, indicating that factors other than age are responsible for the differential anti-glycan IgG and IgM responses. To explore this possibility further, we performed a non-supervised HCA to define other possible individual and glycan clusters.

**Figure 4:**
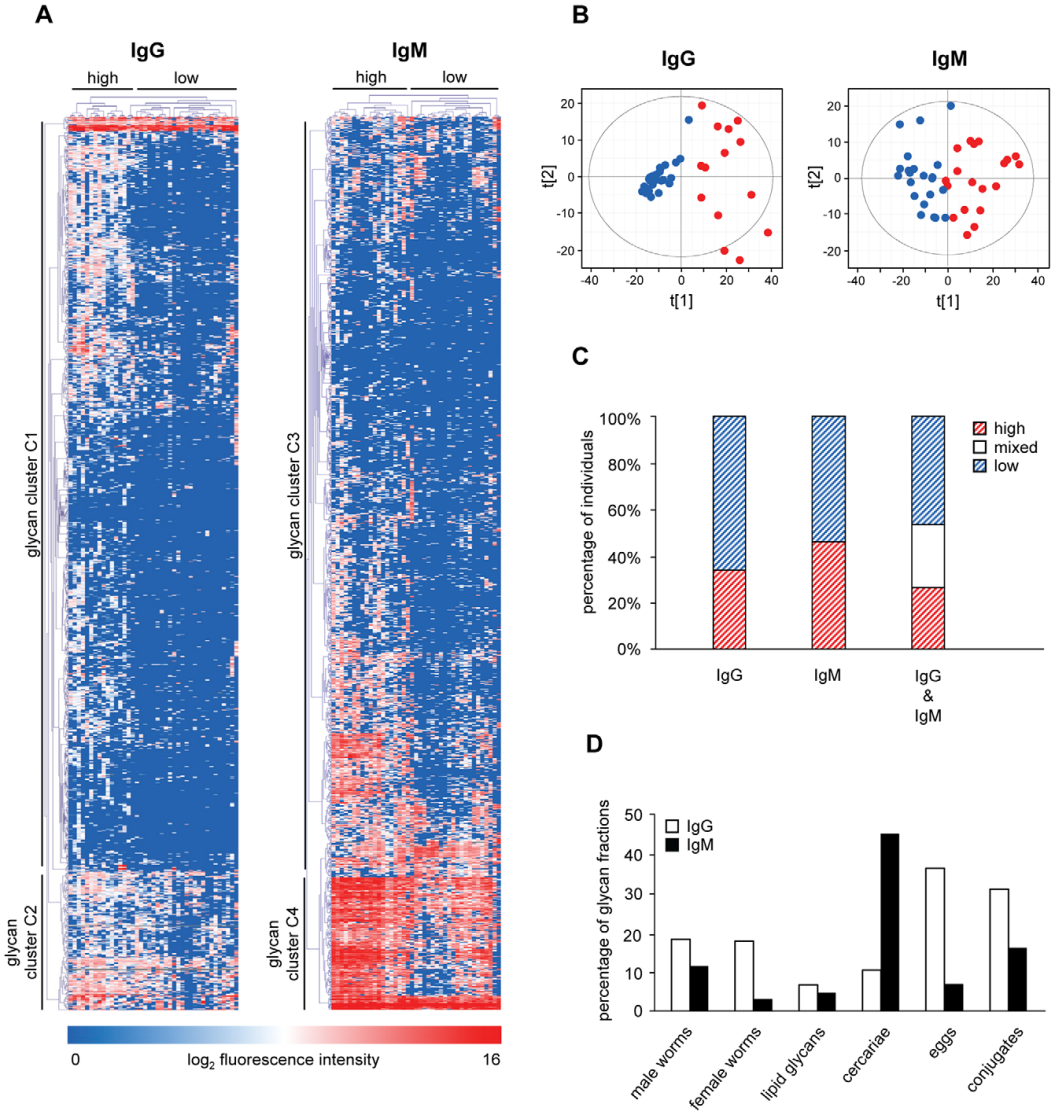
Unsupervised clustering analyses. Hierarchical clustering analysis (A) and PCA (B), percentage of individuals clustering together in the HCA (C) and percentage of glycan fractions in clusters C2 and C4 (D). For HCA, individuals are reported in horizontal dimension and glycan fractions in the vertical dimension. In the PCA, high and low response clusters are represented by red and blue dots, respectively. Percentages of individuals per response cluster in the unsupervised HCA are shown in C. Red and blue bars represent percentage of individuals clustering in high and low response clusters, respectively. White bars represent individuals with low IgG+high IgM and high IgG+low IgM response (mixed). In (D) the number of glycan fractions present in glycan clusters C2 and C4 is depicted as a percentage of the total number of glycan fractions from each source. White and black bars represent results for IgG and IgM, respectively.

Non-supervised HCA and PCA of IgG responses showed two main clusters of individuals with difference in anti-glycan responses (Figure 4A and 4B). The high response cluster 1 contains 14 individuals (12 children and 2 adults) of which 13 were also found in the high response cluster in the supervised age comparisons. The responses for the 27 individuals (9 children and 18 adults) in the other cluster are much lower (Figures 4A, 4B and 4C). With respect to the IgM responses, HCA and PCA also identify a high (red) and a low (blue) response cluster (Figures 4A and 4B). The high response cluster contains 19 individuals (17 children and 2 adults) and 22 individuals fall into the low response group (6 children and 16 adults) (Figure 4D). Although the high response cluster mainly contained children and the low response cluster mainly adults, the non-supervised clustering was different from the supervised clustering on age-dependent differentially expressed glycan fractions indicating that factors other than age play a role in IgM response clustering (data not shown).

From this non-supervised IgG and IgM response analysis for the entire array, four groups of individuals can be defined: group 1 with high IgG and high IgM responses, group 2 with high IgG and low IgM responses (mixed), group 3 with low IgG and high IgM responses (mixed), and group 4 with low IgG and low IgM responses. Group 1 consists of 10 children and 1 adult, while group 4 contains 4 children and 15 adults (Table 1). Interestingly, group 2 (2 children, 1 adult) and group 3 (5 children, 3 adults) do not seem to be biased in terms of age and show intermediate egg counts after treatment.

**Table 1:** Age and egg counts in anti-carbohydrate response clusters.

	response	(group)	n	age	epg1^a^	epg5^a^
IgG	high		14	12.2±2.5	768±197	59±27
	low		27	22.2±2.3	1481±305	11±4
IgM	high		19	13.7±2.6	673±186	55±27
	low		22	23.2±2.3	1726±341	14±6
IgG/IgM	high/high	(1)	11	11.0±2.4	815±197	61±29
	high/low	(2)	3	16.7±8.7	599±305	48±27
	low/high	(3)	8	17.4±5.2	478±186	31±3
	low/low	(4)	19	24.2±2.3	1904±341	8±3

a Eggs per gram of feces at baseline (1) and 9 months (5) post Praziquantel treatment.

Averages ± standard errors are shown.

### Non-supervised comparisons–glycan response clusters within clusters of individuals

The grouping of individuals in the non-supervised HCA and PCA described above was mainly due to glycan clusters C1 and C3 (Figures 4A and 4B) together forming the majority of the glycans present on the shotgun glycan microarray. However, for both IgG and IgM an additional smaller glycan cluster (Figure 4, glycan clusters C2 and C4) was observed for which the grouping of individuals is different. For IgG, glycan cluster C2 mainly consisted of egg (n = 37) and worm N-glycans (n = 35), while IgM glycan cluster C4 mainly contained glycans isolated from cercarial N-glycans (n = 86). When plotting these numbers as percentages of the total number of glycan fractions from each source it was shown that more than one third (36.3%) of the total number of egg-derived N glycans were present in glycan cluster C2 and 44.8% of cercarial N-glycans are present in glycan cluster C4 (Figure 4D).

**Figure 5:**
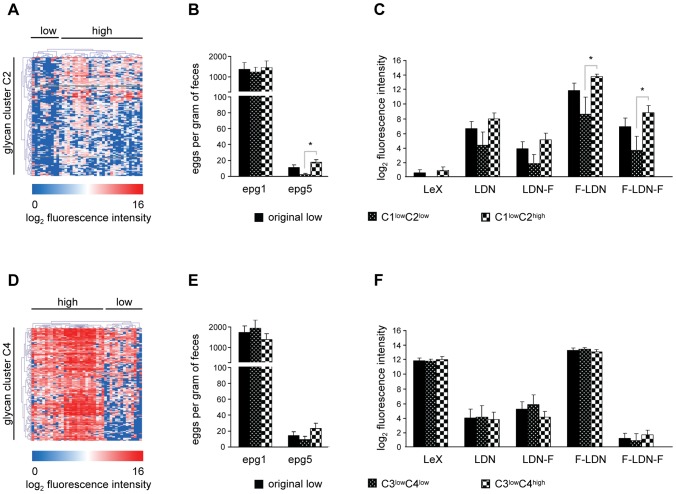
Glycan subclusters analyses. HCA for IgG glycan cluster C2 (A) and IgM glycan cluster C4 (D), egg counts (B and E), and antibody responses against synthetic glycan structures (C and F) in different clusters of individuals. Results are shown for IgG (A–C) and IgM (D–F). Black bars represent individuals in the low response clusters originating from the unsupervised HCA. Black dotted and checkered bars represent individuals from these low response clusters that show a low (C1^low^C2^low^/C3^low^C4^low^) or high response (C1^low^C2^high^/C3^low^C4^high^) for glycan clusters C2 and C4, respectively. Asterisks represent statistical differences between these two groups.

HCA on glycan cluster C2 (Figure 5A) revealed that all individuals from the original high response cluster also belong to the high response group when exploring responses to glycans in cluster C2 only (C1^high^C2^high^). Interestingly, a group of 10 individuals that belonged to the original low response cluster clustered differently from the rest with lower IgG responses against the subset of glycans in glycan cluster C2 (C1^low^C2^low^) and thus differ from the other 17 present in this group of individuals that show an overall low response but show a high response for this selection of glycans in glycan cluster C2 (C1^low^C2^high^). When comparing additional information for these subgroups of individuals from the original low response cluster it became clear that there were no differences in age, but egg counts post treatment (epg5) were lower for those individuals with the lowest IgG responses for glycan cluster C2 (Figure 5B). Strikingly, nine out of ten in the low response cluster were females. The response against the subset of synthetic glycan structures showed that the IgG response in the C1^low^C2^low^ is lower than for the C1^low^C2^high^ group for all glycan structures tested, but significantly lower for F-LDN and F-LDN-F only (Figure 5C).

Also IgM glycan cluster C4 showed a different grouping of individuals than for the complete glycan microarray (Figure 5D). As for IgG, all individuals from the original high response cluster also belong to the high response group when exploring responses to glycans in cluster C4 only (C3^high^C4^high^). However, eight individuals from the original low response cluster show a higher IgM response (C3^low^C4^high^) than the other 14 individuals for the glycans in cluster C4 (C3^low^C4^low^). For this group of 8 individuals the egg counts at 9 months post treatment (epg5) were higher than for the C3^low^C4^low^ group but this was not statistically significant (Figure 5E). In contrast to the clusters of individuals defined by anti-glycan IgG, no differences were observed for the IgM response when comparing C3^low^C4^low^ and C3^low^C4^high^ clusters (Figure 5F).

In particular for IgM responses against the glycans that make up cluster C4 it is clearly visible that the sera fall into three separate groups (Figure 5D), whereas only two groups are observed for cluster C3. This provides an important indication that different subsets of glycans give rise to antibodies which are discriminative for different groups of individuals.

## Discussion

To achieve more insight into the human immune response against *Schistosoma*-derived glycans we analyzed sera of infected individuals for antibody reactivity using a shotgun glycan microarray approach. In this study we selected sera from infected individuals from a larger study in Piida [24] to give two distinct age groups to be compared. In the larger study, *S. mansoni* was found to be highly endemic with an overall prevalence of 72% and with a peak in infection prevalence and intensity in children aged 10–14 years [24], [45], [46]. The selected sera that were chosen allow the exploration of differences in anti-glycan antibody responses between children and adults. In highly schistosomiasis endemic areas like Piida, young children are immunologically, and perhaps physiologically, more susceptible to reinfection after treatment than adults [11], [50] and immunological parameters, including specific antibody and T cell responses, are predictive of the age-dependent immunity or susceptibility to re-infection after treatment [8], [13], [15], [23].

First, we explored the IgG and IgM response to a limited set of synthetic glycoconjugates (Figure 1) to which antibody response profiles have been analyzed previously. In accordance with literature, schistosomiasis induced IgM responses to LeX were higher compared to IgG responses [25], [29], [51]. For GalNAc(β1-4)[Fuc(α1-3)]GlcNAc (LDN-F) high IgM and moderately high IgG responses have been reported [29]. In our glycan microarray analysis this was not the case for children, but when looking at adults only, the relative response to LDN-F is indeed slightly higher for IgM than for IgG (Figure 1). A study on chimpanzees experimentally infected with *S. mansoni* showed that responses to F-LDN and F-LDN-F are similar, and dominated by IgG [51]. Also in our glycan microarray analysis of naturally infected humans, the anti F-LDN-F response is clearly dominated by IgG, but with the response against F-LDN being higher than against F-LDN-F. Specific for the current group comparison of children and adults, significant differences were observed for F-LDN (IgG), F-LDN-F (IgG) and LeX (IgM), with in each case responses being higher in children. One previous study also indicated higher IgG response against F-LDN in children compared to adults living in an endemic area [30]. Just like in the glycan microarray in this study the IgM response against LDN-F was alike in children and adults [30]. Another study showed that median values for the IgM response against LeX were higher in children, but only slightly [25],

While the analysis of antibody responses to the limited set of synthetic conjugates yields some useful insights, the complete glycan microarray includes glycan fractions isolated directly from the schistosome providing the possibility to study numerous additional glycans. Focusing on these glycans, the IgG and IgM responses were also higher in children than adults for most fractions, similar to the observations for the synthetic glycoconjugates. The stronger antibody response against glycans in children and increased susceptibility to reinfection in this age group suggests that there is an inverse correlation between anti-glycan antibody titers and immunity. This would be in line with the smoke screen theory which reasons that high antibody responses towards glycans are beneficial for the parasite rather than the host by subverting the immune system away from epitopes that could provoke protective immune responses [26], [27]. However, anti-glycan antibodies responses cannot be generalized as there are hundreds of different, defined glycan antigens of schistosomes and it could also be hypothesized that while many are subversive, other antibody isotypes or responses to specific subsets of glycan elements may be linked to protective immunity. For example, it has been shown that IgM and IgG2 antibodies that reacted with schistosomula and egg carbohydrate epitopes are negatively associated [14] while IgE directed against glycolipids has been suggested to be positively associated with resistance to reinfection [52]. The glycan clusters in the unsupervised HCA (Figure 4A) also provided an important indication that different subsets of glycans give rise to antibodies which can be discriminative for different groups of individuals and clearly suggested that not all glycans show a similar antibody response.

In the currently used shotgun array, the glycan fractions together contain many different glycan elements expressed by one or more schistosome life stages. While some glycan fractions contain only a single glycan antigen, most fractions are formed of mixtures of glycans that were not separated by the chromatographic procedure used, or they contain glycans which display more than one antigenic glycan element, e.g. in different branches of a di-antennary N-glycan. Therefore, it would be too early to speculate which specific responses to each glycan element occur in the different groups of the cohort. To this end the fractions first need further sub-fractionation and structural analyses to improve separation and definition of the antigenic glycan elements present. What can already be learned from the stage-specific glycan fractions as a group is that for IgG most differentially recognized fractions were derived from cercarial lipid glycans, while IgM responses were clearly most dominant against cercarial N-glycans. Both cercarial lipid and N-glycan fractions contain glycans with LeX elements, however pseudo-Lewis Y elements are unique for cercarial lipid glycans while core β2-xylose occurs in cercarial N-glycans [31], [53], [54] possibly giving rise to differences in dominant responses observed for IgG and IgM. When analyzing the glycan structures in the top 10 of glycan fractions that were differentially recognized between children and adults differences were indeed observed for IgM and IgG. Differential IgM responses between children and adults seem to be mainly against fractions with short fucosylated and/or xylosylated (truncated) core structures and mono LeX elements while differential IgG responses were against more complex structures containing LeX-LeX (dimeric LeX) and LeX-(F-)GlcNAc elements.

The differences in anti-glycan antibody responses between children and adults for this selection of sera may reflect differences in age or differences in length of exposure or infection in an endemic area. The non-supervised HCA showed that the individuals did not cluster precisely according to age suggesting that other factors play a role in anti-glycan antibody response profiles. Also within one cluster of individuals the anti-glycan antibody responses varied for different glycan clusters as was shown for glycan clusters C2 and C4 (Figures 4A and 4B). One single glycan antigen is clearly not representative for the whole group and this stresses the need for screening antibody responses against multiple glycans and glycan elements. Shotgun glycan microarrays are valuable tools in this type of screening allowing the definition of groups of individuals as well as glycan element clusters to which similar antibody responses are generated in individuals. Having shown that the shotgun *Schistosoma* glycan microarray has discriminative power for studying differences in anti-glycan immune responses in different groups of individuals, this technique can now be applied to a randomly-selected epidemiological cohort to address whether anti-glycan antibody responses reflect differences in age, infection intensity or other factors that have not been explored yet.

## Supporting Information

Table S1 (IgG) **Structural information on glycans detected in fractions (top 10) that were differentially recognized between children and adults.**
(TIF)

Table S2 (IgM) **Structural information on glycans detected in fractions (top 10) that were differentially recognized between children and adults.**
(TIF)
