# Supplementary figures and images for: Differential Anti-Glycan Antibody Responses in Schistosoma mansoni-Infected Children and Adults Studied by Shotgun Glycan Microarray
Source: PLoS Negl Trop Dis. 2012 Nov 29;6(11):e1922. doi: 10.1371/journal.pntd.0001922 (PMC3510071; doi:10.1371/journal.pntd.0001922)

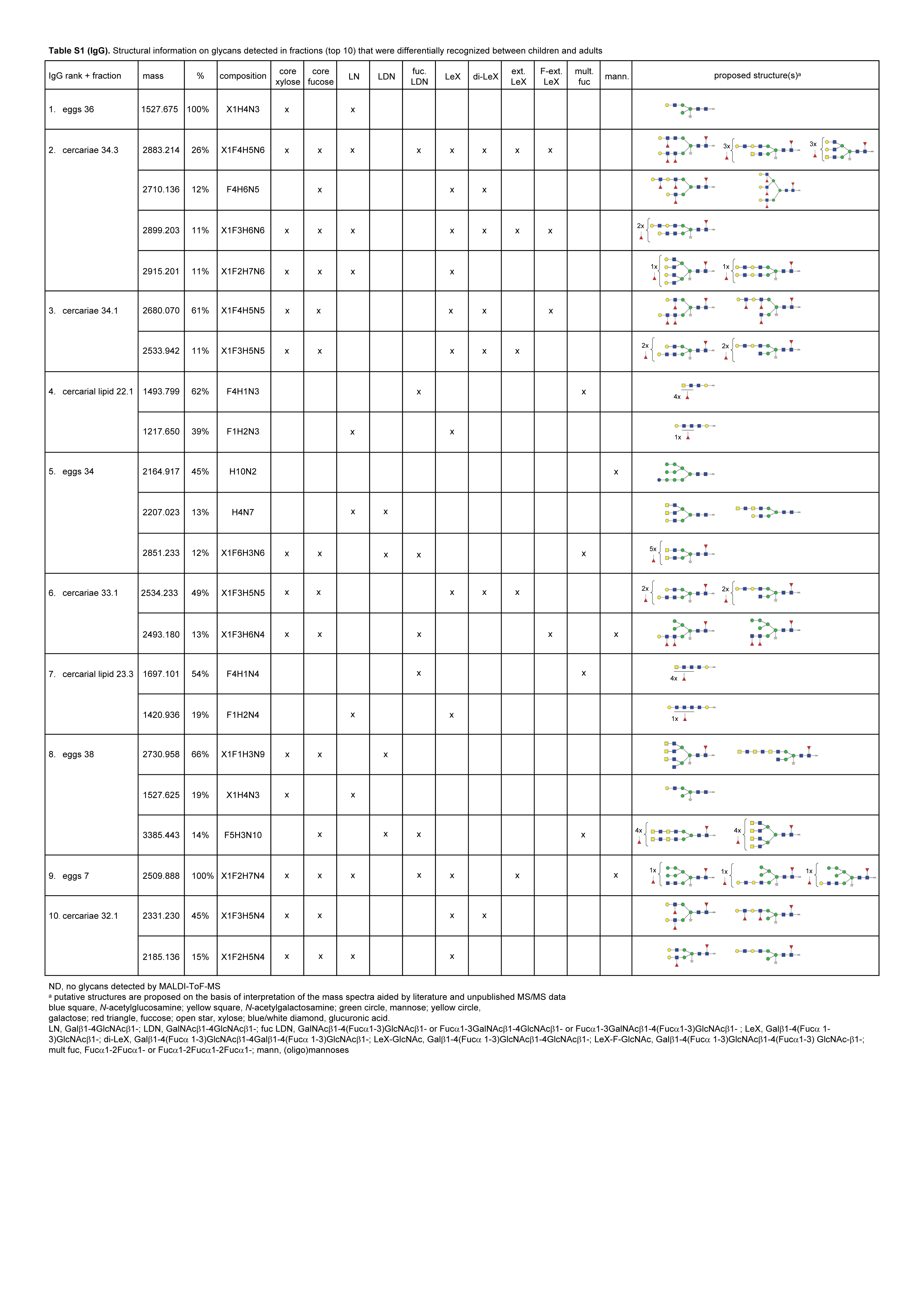

Supplement: Table S1 (IgG) — Structural information on glycans detected in fractions (top 10) that were differentially recognized between children and adults. (TIF) [file pntd.0001922.s001.tif]

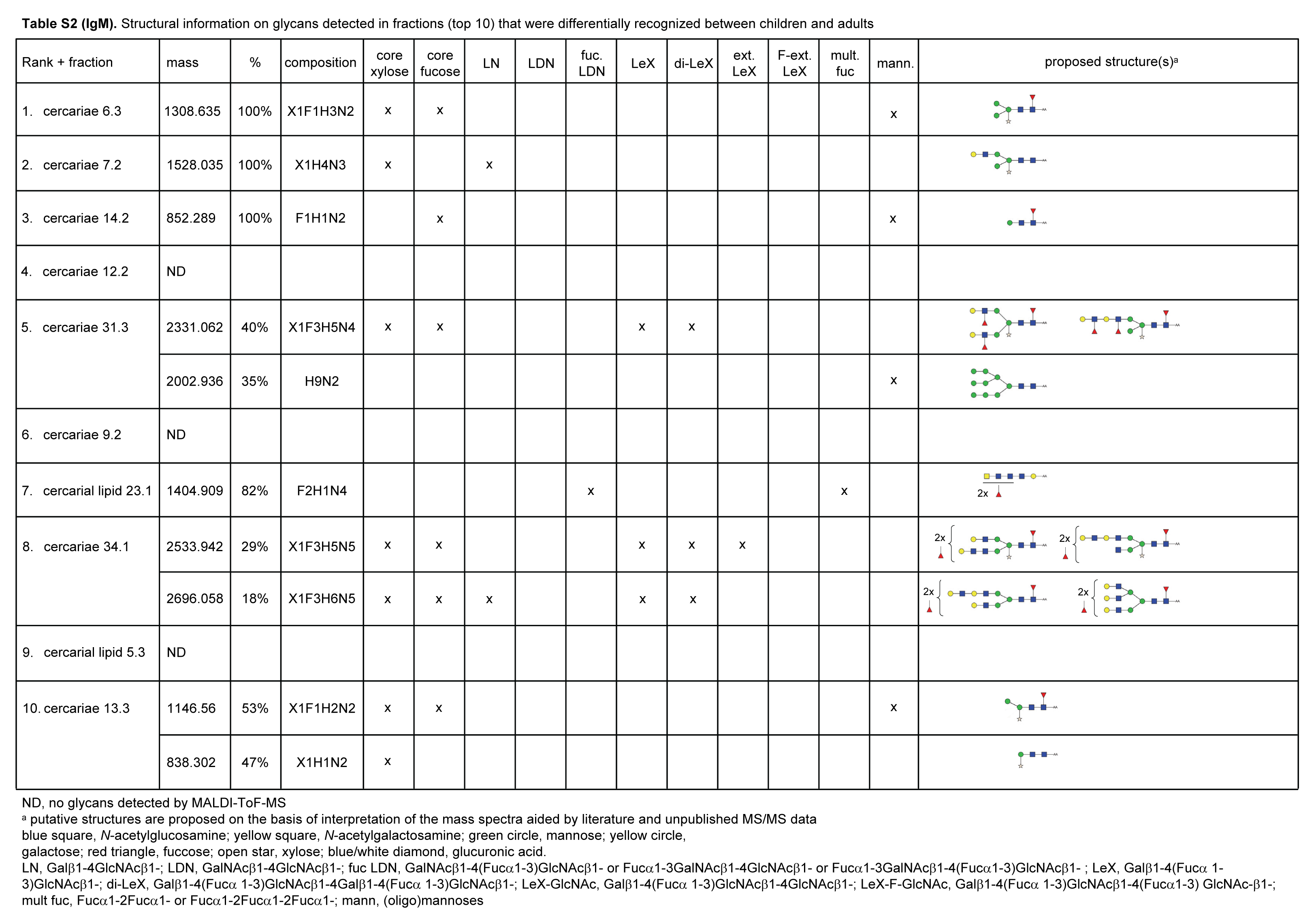

Supplement: Table S2 (IgM) — Structural information on glycans detected in fractions (top 10) that were differentially recognized between children and adults. (TIF) [file pntd.0001922.s002.tif]
